# How do foreign direct investment flows affect carbon emissions in BRICS countries? Revisiting the pollution haven hypothesis using bilateral FDI flows from OECD to BRICS countries

**DOI:** 10.1007/s11356-022-23185-4

**Published:** 2022-09-26

**Authors:** Nicholas Apergis, Mehmet Pinar, Emre Unlu

**Affiliations:** 1 Department of Banking and Financial Management, University of Piraeus, Piraeus, Greece; 2 Business School, Edge Hill University, St Helens Road, Ormskirk, Lancashire UK; 3 Departamento de Análisis Económico y Economía Política, Universidad de Sevilla, Avda. Ramón y Cajal, 1, 41018 Seville, Spain

**Keywords:** Foreign direct investment, Pollution haven hypothesis, Pollution halo effect, OECD countries, BRICS countries

## Abstract

Foreign direct investment (FDI) flows from developed to developing countries may increase carbon emissions in developing countries as developing countries are seen as pollution havens due to their lenient environmental regulations. On the other hand, FDI flows from the developed world may improve management practices and advanced technologies in developing countries, and an increase in FDI flows reduces carbon emissions. Most of the existing studies examine the relationship between FDI flows and carbon emissions by using aggregate FDI flows; however, this paper contributes to the literature by analyzing the impact of FDI flows on carbon emissions in Brazil, Russia, India, China, and South Africa (BRICS) between 1993 and 2012 using bilateral FDI flows from eleven OECD countries. According to our empirical results, from which OECD country FDI flows to BRICS countries matters for carbon emissions in BRICS countries. Our results confirm that FDI flows to BRICS countries from Denmark and the UK increase carbon emissions in BRICS countries, confirming the pollution haven hypothesis. On the other hand, FDI that flows from France, Germany, and Italy reduced carbon emissions in the BRICS countries, confirming the pollution halo effect. FDI flows from Austria, Finland, Japan, Netherlands, Portugal, and Switzerland have no significant impact on carbon emissions in BRICS countries. The BRICS countries should promote clean FDI flows by reducing environmental damages, and investing countries should be rated based on their environmental damage in the host countries.

## Introduction

Economic growth, access to energy, and action for climate change have been some of the priority areas for sustainable development and are integral parts of the United Nations’ Sustainable Development Goals. However, the movement of foreign direct investment (FDI) from developed to developing countries due to less strict environmental laws, cheap labor, and natural resources continue to pressure the climate change goals. Even though FDI flows to the developing countries lead to knowledge spillovers (Branstetter 2006; Xu and Sheng 2012; Paul and Feliciano-Cestero 2021), improved institutional quality in some regions in the host country (Long et al. 2015), and economic growth (Osei and Kim 2020), FDI flows to these countries also increase environmental degradation in developing countries (see, e.g., Hanif et al. 2019, Nawaz et al. 2021; among many others; see also below for further discussion).

The pollution haven hypothesis (PHH) argues that due to weak environmental regulations in host countries, some industries with high contamination and consumption levels will be transferred from other countries through FDI and trade, causing a significant increase in pollutant emissions (see, e.g., Savona and Ciarli 2019; Stef and Jabeur 2020). Most existing studies find a positive association between FDI flows and environmental degradation, which suggest a confirmation of the PHH (see, e.g., Sapkota and Bastola 2017; Hanif et al. 2019; Salehnia et al. 2020; among others). However, recent studies argued that FDI flows could also reduce CO2 emissions due to improvements in management practices and technologies (see, e.g., Zhu et al. 2016; Huang et al. 2017; Wang et al. 2019). FDI flows reducing environmental damage is known as the pollution halo effect (PHE). Finally, a stream of literature found no significant relationship between FDI flows and environmental degradation (see, e.g., Shao et al. 2019; Danish and Ulucak 2022). A detailed summary of more recent literature on the relationship between FDI and environmental quality is presented in Table 1.

**Table 1:** Literature review on PHH and PHE

Literature	Methods	Sample	Period	Findings
Ahmad et al. (2021)	Dynamic common correlated effects mean group method	Chinese provinces	1998–2016	Support for PHE and PHH for different provinces
Assamoi et al. (2020)	Autoregressive distributed lag cointegration (ARDL)	Cote d’Ivoire	1980–2014	Support PHH
Balsalobre-Lorente et al. (2022a)	Dynamic ordinary least squares (DOLS) and fully modified OLS (FMOLS)	BRICS countries	1990–2014	Support PHH
Balsalobre-Lorente et al. (2022b)	DOLS and Dumitrescu-Hurlin causality test	Portugal, Ireland, Italy, Greece, and Spain	1990–2019	Support PHH
Behera and Dash (2017)	Cointegration tests	17 South and Southeast Asian countries	1980–2012	Support PHH
Caetano et al. (2022)	Panel ARDL	15 OECD countries	2005–2018	Support both PHH and PHE for different countries and industries
Cai et al. (2018)	Input–output analysis	66 countries	2013	Support for PHH for some countries, and does not support PHH for some other countries
Chaudhry et al. (2022)	Dynamic common correlated effects (DCCE)	BRICS countries	1995–2019	Support PHH
Djellouli et al. (2022)	Panel ARDL	20 African countries	2000–2015	Support PHH
Gorus and Aslan (2019)	Panel dynamic ordinary least squares	MENA countries	1980–2013	Support PHH
Guzel and Okumus (2020)	CCEMG and augmented mean group (AMG)	Indonesia, Malaysia, Philippines, Singapore, and Thailand	1981–2014	Support PHH
Hanif et al. (2019)	ARDL	15 Asian developing countries	1990–2013	Support PHH
Huang et al. (2017)	Spatial Durbin model	Chinese provinces	2001–2012	Support for PHE
Khan et al. (2020)	Common correlated effect mean group (CCEMG) and fully modified least squares (FM-LS)	BRICS countries	1986–2016	Support PHH
Mahadevan and Sun (2020)	Two-step GMM	China and Belt and 64 Road countries (BRCs)	2003–2014	Support both PHH and PHE
Nasir et al. (2019)	DOLS and FMOLS	ASEAN-5 countries	1982–2014	Support PHH
Nathaniel et al. (2020)	Quantile panel data analysis	10 coastal Mediterranean countries	1980–2016	Support for PHE
Nawaz et al. (2021)	Fully modified ordinary least square (FMOLS)	Pakistan, India, Bangladesh, and Sri Lanka	1990–2018	Support PHH
Pao and Tsai (2011)	Cointegration tests and Granger causality	BRIC countries	1980–2007, 1992–2007 for Russia	Support PHH
Rana and Sharma (2019)	Dynamic multivariate Toda-Yamamoto (TY) approach	India	1982–2013	Support PHH
Ren et al. (2014)	GMM	China	2000–2010	Support PHH
Salahuddin et al. (2018)	Vector error correction model (VECM)	Kuwait	1980–2013	Support PHH
Salehnia et al. (2020)	Panel quantile regression	14 MENA countries	2004–2016	Support for PHE
Sapkota and Bastola (2017)	Fixed and random effects models	14 Latin American countries	1980–2010	Support PHH
Shao et al. (2019)	Vector error correction model (VECM)	BRICS and MINT countries	1982–2014	No significant relationship between FDI and CO2 emissions in BRICS. Support PHE in MINT countries
Singhania and Saini (2021)	GMM and system GMM	21 developed and developing countries	1990–2016	Support for PHE when all countries analyzed; support for PHH when developing countries analyzed
Sun et al. (2017)	ARDL	China	1980–2012	Support PHH
Tamazian et al. (2009)	Fixed and random effects models	BRIC countries	1992–2004	Support for PHE
Tang and Tan (2015)	Vector error-correction model (VECM)	Vietnam	1976–2009	Support for PHE
Wang and Chen (2014)	Two-way fixed-effect models	287 Chinese cities	2002–2009	Support PHH
Wang et al. (2019)	Fixed effects regression model	Beijing-Tianjin-Hebei (BTH) in China	2014–2016	Support for PHE
Waqih et al. (2019)	Panel ARDL and FMOLS	Bangladesh, India, Pakistan, and Sri Lanka	1986–2014	Support PHH
Zakarya et al. (2015)	Cointegration tests and Granger causality	BRICS countries	1990–2012	Support PHH
Zhang and Zhou (2016)	Stochastic impacts by regression on population, affluence, and technology (STIRPAT)	China	1995–2010	Support for PHE
Zhu et al. (2016)	Panel quantile regression	Indonesia, Malaysia, the Philippines, Singapore, and Thailand	1981–2011	Support for PHE
Zhu et al. (2017)	Spatial econometric analysis	Beijing-Tianjin-Hebei region in China	2000–2013	Support PHH

Brazil, Russia, India, China, and South Africa (BRICS) are the fastest growing economies in the world, accounting for 42% and 24% of the world’s population and production (GDP), respectively, and 45% of the world’s CO2 emissions in 2018 (World Bank 2021). Economic growth and heavy reliance on fossil fuels during the production process make BRICS countries the most significant contributors to CO2 emissions and climate change (Chaudhry et al. 2022; Khan et al. 2020; Shao et al. 2019). Therefore, examining the impact of FDI flows on CO2 emissions in BRICS countries is essential.

The existing studies examining the PHH for the BRICS countries employ panel data or time series methods. Those using panel data methods find either support for the PHH (Balsalobre-Lorente et al. 2022a; Chaudhry et al. 2022; Khan et al. 2020; Rana and Sharma 2019; Ren et al. 2014; Wang and Chen 2014; Zakarya et al. 2015) or no support for the PHH (Shao et al. 2019; Danish and Ulucak 2022) or support PHE (see, e.g., Tamazian et al. 2009; Wang et al. 2019). By contrast, PHH is tested using time series or spatial methods for each BRICS country. Using the autoregressive distributed lag cointegration (ARDL) approach, Sun et al. (2017) provide support for the PHH in China. However, Huang et al. (2017) find evidence for the PHE when Chinese provincial data is used through spatial econometric methods. In sum, depending on the methodology employed and the period analyzed, there is a mixed set of findings on the effect of FDI flows on CO2 emissions in BRICS countries.

More recent literature exploring the PHH and PHE aims to explore more disaggregated data by examining provincial data and bilateral trade flows. For example, Ahmad et al. (2021) document that FDI flows heterogeneously influenced CO2 emissions across Chinese provinces. For different regions of China, either PHH or PHE was confirmed, which verifies the presence of aggregation bias. On the other hand, Cai et al. (2018) calculated the carbon emissions due to exports and imports in China and highlighted that China had become a pollution haven for 22 developed countries, while 19 developing countries have become China’s pollution haven. As highlighted by Ahmad et al. (2021), the studies that employ aggregate FDI flows in their analysis may suffer from aggregation bias. Therefore, this study examines the impact of FDI flows on CO2 emissions in BRICS countries between 1993 and 2012 using bilateral FDI flows from eleven OECD countries to BRICS countries.

This paper aims to contribute to the literature in various ways. Firstly, rather than using aggregate FDI flows to examine the impact of FDI flows on carbon emissions in BRICS countries, we use disaggregated FDI flows to investigate whether there is any support for PHH or PHE depending on which OECD country FDI flows to BRICS counties. For instance, Sun et al. (2017) illustrate that China is a pollution haven when the aggregate FDI flows to China are examined. However, it is possible that the FDI flows from some countries may lead to technological improvements and confirm PHE. Alternatively, some other countries may consider BRICS countries as pollution havens and FDI flows from these countries may increase CO2 emissions in BRICS countries. Therefore, we overcome the potential aggregation bias by using bilateral FDI flows to BRICS countries. Secondly, by using disaggregated FDI flows data, we can shed light on why existing studies may have found mixed results about the impact of FDI flows on CO2 emissions in BRICS countries. Thirdly, some of the existing studies do not tackle the potential endogeneity problem. However, endogeneity is a serious concern as some studies found that CO2 emissions in BRICS countries may lead to higher FDI flows (e.g., Shao et al. 2019). Therefore, this paper employs a general method of moments (GMM) to account for potential endogeneity and does not suffer from biases that arise due to the endogeneity problem.

The remainder of the paper is organized as follows. “Methodology” section provides details of the methodology and data. “Empirical analysis” section offers the empirical findings, and “Robustness analysis” section provides robustness analysis. Finally, “Conclusions and policy implications” section provides conclusions and policy recommendations.

## Methodology

### Model and variables

The goal is to explore the role of net FDI inflows from each selected OECD country in explaining the CO2 emissions in the BRICS countries. The model specification yields:

1 $${CO2}_{i,t}=a\;{CO2}_{i,t-1}+b\;{FDI}_{i,t}+c_1{GDPY}_{i,t}+c_2{\;ENUSE}_{i,t}+c_3{\;TR}_{i,t}+c_4\;{POP}_{i,t}+c_5{\;URBPOP}_{i,t}+c_6{\;REN}_{i,t}+\alpha_i+\beta_t+v_{i,t}$$

where CO2 is carbon emissions per capita, FDI denotes net FDI inflows from each OECD country to the BRICS countries, GDPY is GDP per capita, ENUSE shows energy use, TR denotes trade activities, POP is total population, URBPOP denotes urban population, and REN is renewable energy consumption. The model also accounts for country and time fixed effects, *α*_*i*_ and *β*_*t*_, respectively. In a panel framework, the error terms, ${v}_{i,t}$, are uncorrelated. They are assumed to be independently distributed across countries with a zero mean. To avoid the presence of potential endogeneity issues, we estimate the dynamic panel data model using the general method of moments (GMM) estimation recommended by Arellano and Bover (1995) and Blundell and Bond (1998). The presence of endogeneity potentially could come through reverse causality between carbon emissions and any of the covariates. For instance, Shao et al. (2019) found that the CO2 emissions in BRICS countries explain the FDI flows and trade openness using the vector error correction model. Al-Mulali and Ozturk (2016) also found that the relationship between CO2 emissions and GDP per capita is bidirectional. Along the same lines, Tang and Tan (2015) found bidirectional causation between CO2 emissions and energy consumption in Vietnam. Overall, the existing literature found reverse causality between carbon emissions and other covariates. Therefore, we use the GMM estimation method to tackle potential endogeneity problems (see also He 2006; Du et al. 2012; Ren et al. 2014; Li et al. 2016; Hove and Tursoy 2019; Mahadevan and Sun 2020; Singhania and Saini 2021, among others, for the use of GMM methods to overcome endogeneity problem when pollution is used as a dependent variable). In addition, the empirical analysis will use a non-causality test developed by Dumitrescu and Hurlin (2012). This test can be used when *T* > *N* (with *T* being the number of observations and *N* the number of countries considered), which is our case here. The corresponding Wald statistic is defined as follows: *Z*_*N*,*T*_ = √*N*/2* K* (*W*_*N*,*T*_ − *K*), where *K* is the number of lags in the corresponding VAR model, and:

2 $${W}_{N,T}=1/N\sum_{i=1}^{N}{W}_{i,T}$$

where *W*_*i*,*T*_ stands for the individual Wald statistical values for cross-section units.

### Data

The data for all the variables, except bilateral FDI flows, are obtained from the World Development Indicators (World Bank 2021). The bilateral FDI flows from eleven OECD to BRICS countries are obtained from OECD (2021) and are measured in US dollars. Bilateral FDI flows per capita are obtained by dividing aggregate bilateral FDI flows by the total population. The bilateral FDI flows data is available between 1985 and 2013; however, most data before 1993 and 2013 had missing values. Therefore, our analysis covers the period between 1993 and 2012 to capture as many OECD countries as possible in the study. The OECD countries used in this analysis are Austria, Denmark, Finland, France, Germany, Italy, Japan, Netherlands, Portugal, Switzerland, and the UK.

As a determinant of environmental degradation, the existing literature accounts for the population size (see, e.g., Wang et al. 2015; Zhu et al. 2016), urbanization (see, e.g., Al-Mulali and Ozturk 2015; Anwar et al. 2022; Chien et al. 2022; Dong et al. 2020; Hossain 2011; Murshed et al. 2021; Nadeem et al. 2020; Sarkodie and Ozturk 2020), trade openness (see, e.g., Al-Mulali and Ozturk, 2015; Hossain 2011; Kolcava et al. 2019; Le et al. 2016; Lin, 2017; Shahbaz et al., 2017; Zhang, 2020), energy consumption and renewable energy consumption (see, e.g., Ambe 2021; Charfeddine and Kahia 2019; Chen et al. 2022; Godil et al. 2020; Rahman et al. 2022; Shahnazi and Shabani 2021; Sharif et al. 2019; Usman and Balsalobre-Lorente 2022), and economic development or growth (see, e.g., Doğan et al. 2022; Jahanger et al. 2022; Karahasan and Pinar 2022; Sharif et al. 2020a; Sharif et al. 2020b; Suki et al. 2020).

As our dependent variable, we use CO2, which is carbon dioxide emissions (measured in metric tons) per capita. We also use the following independent variables in our study. FDI denotes net FDI inflows per capita from the OECD country to the BRICS countries. GDPY is GDP per capita (constant 2010 US$), ENUSE is the total energy consumption (measured in kg of oil equivalent) per capita. TR is the trade openness measure, which is the sum of exports and imports as a percentage of GDP. POP is the total population of the respective BRICS country. URBPOP is the urban population and is measured as the percentage of the population living in the urban areas. Finally, REN is the renewable energy consumption, which is measured as the renewable energy consumption as a percentage of total final energy consumption.

Table 2 offers the descriptive statistics for each variable and net FDI flows per capita from each OECD country to BRICS countries. For a given average year and BRICS country, net FDI flows per capita to BRICS countries were higher from the UK, France, Japan, and Germany (i.e., $1255, $426, $380, and $355, respectively), and were lower from Denmark, Finland, and Portugal (i.e., $27, $42, and $61, respectively).^1^ The urbanization rates also showed an increasing trend in each BRICS country. The percentages of the urban population were 76%, 73%, 26%, 29%, and 54% in 1993, and were 85%, 74%, 32%, 52%, and 63% in 2012 in Brazil, India, Russia, China, and South Africa, respectively. Similarly, there has been an increasing trend in trade openness across all the BRICS countries. Renewable energy as a percentage of the total final energy consumption decreased in all of the BRICS countries between 1993 and 2012. The percentages of renewable energy consumption in the energy mix were 48%, 57%, 4%, 32%, and 19% in 1993, and were 44%, 39%, 3%, 12%, and 11% in 2012 in Brazil, India, Russia, China, and South Africa, respectively. Furthermore, there has been a clear increasing trend in the CO2 emissions per capita, GDP per capita, and energy consumption between 1993 and 2012.

**Table 2:** Descriptive statistics

	Mean	Standard deviation	Minimum	Maximum
FDI flows from Austria	96.5	376.3	− 547.7	2606.4
FDI flows from Denmark	27.1	78.3	− 370.7	530.9
FDI flows from Finland	41.5	131.2	− 304.2	747.4
FDI flows from France	425.5	828.5	− 764.9	4866.2
FDI flows from Germany	354.6	1070.5	− 3362.2	6968.0
FDI flows from Italy	129.8	311.3	− 394.9	2083.9
FDI flows from Japan	379.9	611.7	− 47.4	4144.5
FDI flows from the Netherlands	260.0	724.8	− 1842.4	5212.9
FDI flows from Portugal	60.6	356.8	− 1220.6	2675.5
FDI flows from Switzerland	298.9	880.0	− 479.5	6930.7
FDI flows from the UK	1254.8	3061.9	− 2010.1	16,583.7
CO2 emissions (metric tons per capita)	4.9	3.7	0.7	12.3
GDP per capita (constant 2010 US$)	5522.0	3501.1	611.1	11,745.8
Energy use (kg of oil equivalent per capita)	1993.8	1470.5	364.5	5167.0
Trade openness	42.5	15.2	15.6	72.9
Population	549,732,129.1	531,726,681.9	39,633,754.0	1,350,695,000.0
Urban population	56.3	20.3	26.2	84.9
Renewable energy consumption	26.9	18.3	3.2	56.9

Total number of observations is 100

## Empirical analysis

This section examines the causal relationship between net FDI flows from each OECD country to BRICS countries and the CO2 emissions in BRICS countries. However, we first need to conduct some tests to identify the presence of cross-sectional dependence. Studies examining the factors contributing to CO2 emissions have extensively considered the potential cross-section dependence before their analysis (Churchill et al. 2018; Belaïd and Zrelli 2019; Dogan et al. 2020; Munir et al. 2020, among others). Therefore, we first explore the degree of residual cross-sectional dependence through the cross-sectional dependence (CD) statistic proposed by Pesaran (2004). The results are reported in Table 3, and we reject the null hypothesis of cross-sectional independence for all the variables.

**Table 3:** Cross dependence tests

Variables	CD test	*p* value
CO2	5.83	0.01
FDI Austria	7.13	0.00
FDI Denmark	5.96	0.00
FDI Finland	6.11	0.00
FDI France	6.64	0.00
FDI Germany	7.39	0.00
FDI Italy	6.42	0.00
FDI Japan	6.59	0.00
FDI Netherlands	7.12	0.00
FDI Portugal	6.52	0.00
FDI Switzerland	6.69	0.00
FDI UK	7.16	0.00
GDPY	6.43	0.00
ENERGYUSE	6.58	0.00
TRADE	7.39	0.00
POP	6.40	0.00
URBPOP	6.46	0.00
REN	6.18	0.00

The test is based on the sum of correlation coefficient squares among cross-sectional residuals. This test, which is asymptotically standard normal distribution, examines the null hypothesis of cross-sectional independence

Next, a second-generation panel unit root test, the Pesaran (2007) panel unit root test, is used to determine the degree of integration of the respective variables. The null hypothesis suggests a presence of a unit root. The results are reported in Table 4 and support the presence of a unit root across all variables, and the non-stationarity of these variables in their first differences is rejected. Moreover, concerning the FDI flows from each OECD country to BRICS countries, the unit root test of the generalized least squares (GLS), recommended by Elliott et al. (1996), is used and the results are shown in Table 4. The findings indicate that the respective variables are stationary in their levels.

**Table 4:** Unit root tests

Variables	CIPS tests	
	Levels	1st differences
CO2	− 1.485	− 6.419***
GDPY	− 1.602	− 6.336***
ENERGYUSE	− 1.649	− 6.782***
TRADE	− 1.132	− 8.503***
POP	− 1.282	− 6.235***
URBPOP	− 1.064	− 6.537***
REN	− 1.095	− 6.882***
	GLS test—levels	
FDI Austria	− 3.067(3)***	
FDI Denmark	− 3.889(3)***	
FDI Finland	− 3.620(2)***	
FDI France	− 3.542(4)***	
FDI Germany	− 3.582(4)***	
FDI Italy	− 3.223(3)***	
FDI Japan	− 3.788(3)***	
FDI Netherlands	− 3.764(4)***	
FDI Portugal	− 5.757(3)***	
FDI Switzerland	− 4.062(2)***	
FDI UK	− 6.605(4)***	

A constant is included in the Pesaran (2007) test. Critical values are − 2.40, − 2.22, and − 2.14 at the 1%, 5%, and 10% levels, respectively. The Pesaran results are reported at lag 4. Figures in parentheses with respect to the GLS test denote the number of lags included and the numbers of lags used were determined through the Akaike information criterion. ***p ≤ 0.01

Table 5 reports the baseline empirical results of the static GMM model. The regression analysis includes the same control variables (i.e., GDPY, ENERGYUSE, TRADE, POP, URBPOP, REN) and lagged CO2 emissions per capita in the BRICS countries. The only variable that varies across different columns of Table 5 is the FDI inflows per capita from each OECD country to the BRICS countries. Columns 1–11 report the FDI flows from the respective OECD countries to the BRICS countries. The estimates document a negative and statistically significant impact of FDI flows on CO2 emissions in the BRICS countries if the FDI flows are from France, Germany, and Italy. The technical effect of the FDI flows from these countries (i.e., better management practices and environment-friendly technologies used in the production) dominates the factor endowment effects (Zugravu-Soilita 2017). However, the impact of FDI flows on CO2 emissions is positive and statically significant if FDI flows are from Denmark and the UK. The finding concerning FDI flows from the UK is in line with that recommended in the literature. Mulatu (2017) examines the UK-based multinational activity in 64 countries over the period 2002–2006 and finds that FDI flows from the UK target relatively more polluting industries in countries with lax environmental regulations. Finally, the effect of the FDI flows from Austria, Finland, Japan, Netherlands, Portugal, and Switzerland on CO2 emissions is statistically insignificant. These findings explain why current literature finds mixed results concerning the effect of FDI flows on environmental degradation in the BRICS countries. As the existing research papers use aggregate FDI flows in their analysis, they suffer from aggregation bias (Ahmad et al. 2021).

**Table 5:** CO2 and FDI: GMM estimates (the static model)

	(1)	(2)	(3)	(4)	(5)	(6)	(7)	(8)	(9)	(10)	(11)
	Austria	Denmark	Finland	France	Germany	Italy	Japan	Netherlands	Portugal	Switzerland	UK
CO2(− 1)	0.603***	0.597***	0.594***	0.622***	0.581***	0.591***	0.566***	0.571***	0.558***	0.586***	0.619***
	[0.00]	[0.00]	[0.00]	[0.00]	[0.00]	[0.00]	[0.00]	[0.00]	[0.00]	[0.00]	[0.00]
FDI FLOWS	− 0.0458	0.0088*	− 0.0116	− 0.0236***	− 0.0339**	− 0.0756***	0.00814	0.000145	− 0.00007	− 0.00020	0.0268***
	[0.15]	[0.09]	[0.16]	[0.00]	[0.04]	[0.00]	[0.44]	[0.73]	[0.81]	[0.44]	[0.00]
$\Delta$ GDPY	0.0173**	0.0078*	0.0147**	0.0195**	0.0102*	0.0208***	0.00785	0.000618	0.000387	0.000189	0.0148*
	[0.05]	[0.10]	[0.05]	[0.03]	[0.10]	[0.00]	[0.22]	[0.43]	[0.60]	[0.89]	[0.06]
$\Delta$ ENERGYUSE	0.0219***	0.0204***	0.0214***	0.0211***	0.0211***	0.0219***	0.0210***	0.0257***	0.0256***	0.0256***	0.0251***
	[0.00]	[0.00]	[0.00]	[0.00]	[0.00]	[0.00]	[0.00]	[0.00]	[0.00]	[0.00]	[0.00]
$\Delta$ TRADE	0.0423***	0.0462***	0.0451***	0.0448***	0.0467***	0.0447***	0.0469***	0.00185	0.00192	0.00342	0.0103*
	[0.00]	[0.00]	[0.00]	[0.00]	[0.00]	[0.00]	[0.00]	[0.80]	[0.80]	[0.97]	[0.07]
$\Delta$ POP	0.00728	0.00383	0.00217	0.00176	0.00289	0.00136	0.00348	0.00042	0.00042	0.00040	0.0114**
	[0.11]	[0.37]	[0.49]	[0.57]	[0.37]	[0.66]	[0.25]	[0.48]	[0.49]	[0.51]	[0.04]
$\Delta$ URBPOP	0.0409***	0.0301***	0.0408***	0.0415***	0.0325***	0.0460***	0.0302***	0.0214*	0.0209	0.0230	0.0135**
	[0.00]	[0.00]	[0.00]	[0.00]	[0.00]	[0.00]	[0.00]	[0.10]	[0.52]	[0.48]	[0.05]
ΔREN	− 0.0784***	− 0.0912***	− 0.0904***	− 0.0813***	− 0.0844***	− 0.0693***	− 0.0688***	− 0.0715***	− 0.0674***	− 0.0655***	− 0.0724***
	[0.00]	[0.00]	[0.00]	[0.00]	[0.00]	[0.00]	[0.00]	[0.00]	[0.00]	[0.00]	[0.00]
Diagnostics											
Country fixed effects	Yes	Yes	Yes	Yes	Yes	Yes	Yes	Yes	Yes	Yes	Yes
Time fixed effects	Yes	Yes	Yes	Yes	Yes	Yes	Yes	Yes	Yes	Yes	Yes
*R*^2^-adjusted	0.88	0.89	0.91	0.94	0.88	0.92	0.78	0.69	0.68	0.63	0.81
AR(2)	[0.46]	[0.45]	[0.51]	[0.54]	[0.59]	[0.59]	[0.43]	[0.40]	[0.39]	[0.38]	[0.50]
Hansen overidentification	[0.99]	[0.99]	[0.99]	[0.99]	[0.99]	[0.99]	[0.99]	[0.99]	[0.99]	[0.99]	[0.99]
Difference-in-Hansen	[0.99]	[0.99]	[0.99]	[0.99]	[0.99]	[0.99]	[0.98]	[0.97]	[0.94]	[0.93]	[0.98]

Following Roodman (2009), GMM instruments are collapsed to avoid over-proliferation. AR(2) is the test for auto-correlation of order 2 in first-differenced errors. Difference-in-Hansen is the test of the validity of GMM instruments. The null hypothesis is that the instruments are valid (i.e., uncorrelated with the error term). *p ≤ 0.10, **p ≤ 0.05, ***p ≤ 0.01

Regarding the remaining determinants of CO2 emissions in BRICS countries, the estimates document that income per capita, energy use, trade, and urban population exert a positive and statistically significant impact on CO2 emissions across most of the specifications. However, the population size is insignificant in most of the specifications. These findings are in line with the current literature. Lagged CO2 emissions are positive, which is the case for the literature using the GMM estimation methods (Ren et al. 2014; Li et al. 2016; Hove and Tursoy 2019; Singhania and Saini 2021). CO2 emissions increase with the increased GDP per capita (Baloch et al. 2020; Chaudhry et al. 2022; Ren et al. 2014; Shao et al. 2019), trade openness (Rana and Sharma 2019; Ren et al. 2014), increased energy consumption (Chaudhry et al. 2022; Khan et al. 2020; Li et al. 2016), increased urbanization (Al-Mulali and Ozturk 2015; Anwar et al. 2022; Behera and Dash 2017; Murshed et al. 2021; Nadeem et al. 2020), and decreased renewable energy consumption (Balsalobre-Lorente et al. 2022b, a; Djellouli et al. 2022; Murshed et al. 2021; Sharif et al. 2019).

Finally, specific diagnostics are also reported in Table 5. The AR(2) test results suggest that the null hypothesis is rejected, indicating no second-order serial correlation. Furthermore, difference-in-Hansen is the test of the validity of GMM instruments. The difference-in-Hansen test rejects the null hypothesis, and therefore findings support the validity of the instruments used.

## Robustness analysis

### The dynamic model

This section repeats the baseline analysis, but the dynamic version of Eq. (1) is considered in which certain lags of the controls covariates are used in the estimation. The number of lags is determined through the Akaike criterion. The new findings are reported in Table 6 and provide robust support to those reported previously. The analysis in Table 6 also includes the first lags of the FDI flows, GDP per capita, and trade openness in certain specifications. Lagged FDI flows from Denmark and the UK lead to increased CO2 emissions, confirming the PHH. The coefficients of lagged FDI flows from France, Italy, and Germany are also negative and statistically significant, confirming the PHE. In contrast, the remaining results align with the ones reported in Table 5. Finally, the diagnostics tests confirm the validity of the instruments.

**Table 6:** CO2 and FDI: GMM estimates (the dynamic model)

	(1)	(2)	(3)	(4)	(5)	(6)	(7)	(8)	(9)	(10)	(11)
	Austria	Denmark	Finland	France	Germany	Italy	Japan	Netherlands	Portugal	Switzerland	UK
CO2(− 1)	0.552***	0.596***	0.564***	0.570***	0.556***	0.549***	0.543***	0.514***	0.534***	0.546***	0.607***
	[0.00]	[0.00]	[0.00]	[0.00]	[0.00]	[0.00]	[0.00]	[0.00]	[0.00]	[0.00]	[0.00]
FDI	− 0.0385	0.0072*	− 0.0103	− 0.0211***	− 0.0304**	− 0.0708***	0.00728	0.00009	− 0.00006	− 0.00014	0.0249***
	[0.22]	[0.10]	[0.17]	[0.01]	[0.05]	[0.00]	[0.57]	[0.77]	[0.82]	[0.51]	[0.01]
FDI(− 1)	− 0.0373	0.0052	− 0.0078	− 0.0153**	− 0.0286*	− 0.0626**		0.000092		− 0.00012	0.0213**
	[0.24]	[0.12]	[0.20]	[0.03]	[0.06]	[0.03]		[0.80]		[0.53]	[0.04]
$\Delta$ GDPY	0.0138*	0.0071	0.0134*	0.0168**	0.0091*	0.0201***	0.0055	0.000526	0.000339	0.000161	0.0136*
	[0.06]	[0.11]	[0.06]	[0.04]	[0.10]	[0.01]	[0.28]	[0.48]	[0.66]	[0.91]	[0.06]
$\Delta$ GDPY(− 1)	0.0129*			0.0106*	0.0057		0.00281	0.000486			0.0102*
	[0.07]			[0.06]	[0.12]		[0.40]	[0.57]			[0.09]
$\Delta$ ENERGYUSE	0.0184***	0.0185***	0.0201***	0.0189***	0.0189**	0.0193***	0.0184**	0.0219***	0.0226***	0.0229***	0.0238***
	[0.00]	[0.00]	[0.00]	[0.00]	[0.02]	[0.00]	[0.02]	[0.01]	[0.01]	[0.00]	[0.01]
$\Delta$ TRADE	0.0403***	0.0419***	0.0392***	0.0410***	0.0416***	0.0405***	0.0411**	0.0012	0.00171	0.00307	0.0095*
	[0.00]	[0.00]	[0.00]	[0.00]	[0.00]	[0.00]	[0.02]	[0.84]	[0.81]	[0.98]	[0.08]
$\Delta$ TRADE(− 1)	0.0372**	0.0324**	0.0368**	0.0346**	0.0329**	0.0328**	0.0357**		0.00138		
	[0.02]	[0.04]	[0.02]	[0.03]	[0.04]	[0.03]	[0.03]		[0.84]		
$\Delta$ POP	0.00685	0.00238	0.00195	0.00124	0.00250	0.00115	0.00308	0.00036	0.00031	0.00031	0.0102**
	[0.11]	[0.42]	[0.53]	[0.61]	[0.41]	[0.69]	[0.30]	[0.53]	[0.56]	[0.55]	[0.05]
$\Delta$ URBPOP	0.0394***	0.0283***	0.0370***	0.0387***	0.0296***	0.0403***	0.0266**	0.0194	0.0185	0.0209	0.0124*
	[0.00]	[0.01]	[0.00]	[0.00]	[0.00]	[0.00]	[0.02]	[0.11]	[0.56]	[0.50]	[0.07]
REN	− 0.0675***	− 0.0784***	− 0.0766***	− 0.0712***	− 0.0688***	− 0.0613***	− 0.0574***	− 0.0609***	− 0.0564***	− 0.0624***	− 0.0684***
	[0.00]	[0.00]	[0.00]	[0.00]	[0.00]	[0.00]	[0.00]	[0.00]	[0.00]	[0.00]	[0.00]
REN(− 1)	− 0.0374**		− 0.0386**	− 0.0321**				− 0.0246**	− 0.0209**		− 0.0306**
	[0.03]		[0.02]	[0.03]				[0.04]	[0.05]		[0.03]
Diagnostics											
Country fixed effects	Yes	Yes	Yes	Yes	Yes	Yes	Yes	Yes	Yes	Yes	Yes
Time fixed effects	Yes	Yes	Yes	Yes	Yes	Yes	Yes	Yes	Yes	Yes	Yes
*R*^2^-adjusted	0.91	0.89	0.94	0.96	0.890	0.93	0.81	0.68	0.65	0.56	0.79
AR(2)	[0.52]	[0.49]	[0.57]	[0.58]	[0.61]	[0.64]	[0.49]	[0.39]	[0.39]	[0.37]	[0.51]
Hansen overidentification	[0.99]	[0.99]	[0.99]	[0.99]	[0.99]	[0.99]	[0.99]	[0.99]	[0.99]	[0.99]	[0.99]
Difference-in-Hansen	[0.99]	[0.99]	[0.99]	[0.99]	[0.99]	[0.99]	[0.99]	[0.96]	[0.97]	[0.92]	[0.99]

Following Roodman (2009), GMM instruments are collapsed to avoid over-proliferation. AR(2) is the test for auto-correlation of order 2 in first-differenced errors. The difference-in-Hansen is the test of the validity of GMM instruments. The null hypothesis is that the instruments are valid (i.e., uncorrelated with the error term). *p ≤ 0.10, **p ≤ 0.05, ***p ≤ 0.01

### Panel non-causality test

The panel non-causality test developed by Dumitrescu and Hurlin (2012) is performed in this part. Under the null hypothesis, it is assumed that there is no causality from one variable to another. Under the alternative hypothesis, there exists a causal relationship from one variable to another only for a subgroup of individuals, with the coefficients differing across groups.

The causality results are reported in Table 7. The cases of FDI flows from Austria, Denmark, Finland, France, Germany, Italy, and the UK document univariate causality from FDI flows to CO2 emissions. For the remaining cases of FDI flows from the rest of the OECD countries, the non-causality hypothesis is accepted. In other words, the findings confirm the results obtained with the static and dynamic GMM models. In other words, our findings confirm the PHH hypothesis if FDI flows are from Denmark and the UK, and the PHE hypothesis if FDI flows are from France, Germany, and Italy.

**Table 7:** Dumitrescu and Hurlin panel Granger non-causality test results

Null hypothesis	*p* value
*FDI does not cause carbon emissions*	
Austria	[0.00]
Denmark	[0.00]
Finland	[0.00]
France	[0.00]
Germany	[0.00]
Italy	[0.00]
Japan	[0.17]
Netherlands	[0.18]
Portugal	[0.22]
Switzerland	[0.26]
UK	[0.00]
*Carbon emissions does not cause FDI*	
Austria	[0.17]
Denmark	[0.20]
Finland	[0.14]
France	[0.17]
Germany	[0.21]
Italy	[0.25]
Japan	[0.28]
Netherlands	[0.20]
Portugal	[0.23]
Switzerland	[0.25]
UK	[0.19]

## Conclusions and policy implications

There has been an increased debate on the PHH and PHE to examine the effect of FDI flows on the environmental quality in recipient countries, with much of the research exploring the role of different characteristics of the recipient countries. However, the role of the investing countries on the environment quality in the recipient countries has received little attention. This paper contributes to the existing literature by examining the effect of FDI flows from eleven OECD countries on CO2 emissions in the BRICS countries. We found that from which country FDI flows matter for the environmental degradation in recipient countries. The findings suggested that while FDI flows from Denmark and the UK led to increased CO2 emissions in the BRICS countries, FDI flows from France, Germany, and Italy decreased CO2 emissions in these countries. The findings were robust to the estimation model selection (i.e., static and dynamic GMM estimation method and panel Granger non-causality test). Overall, the results highlighted that the FDI flows coming from different OECD countries had a heterogeneous effect on the environmental quality in the BRICS countries, and the importance of examining the PHH and PHE by using the disaggregated data as the aggregated data may shadow some of the existing mechanisms in place.

The findings of this paper have important policy implications. Both investing and recipient countries have their roles to play in combating climate change. More specifically, the recipient countries should adjust their degree of stringency of environmental regulations disallowing themselves to be “pollution havens” and more so if the FDI flows from a set of countries. Furthermore, beyond the performance of the recipient countries, international agencies could publish a rating system of the investing countries based on the environmental performance of their investors abroad. This concept is closely associated with the donor ratings in international aid effectiveness literature (see, e.g., Roodman 2012; Minasyan et al. 2017). Providing such a rating system may expose investors that target other countries as pollution havens and could pressure these investors to alter their behavior and reduce the negative implications of their investments in recipient countries.

This paper has some limitations. Firstly, our empirical analysis covers FDI flows from each of the eleven OECD countries to BRICS countries to examine the effect of FDI flows on CO2 emissions in BRICS countries between 1993 and 2012. In other words, the empirical analysis of this paper has limited country and period coverage. Future studies could expand the country coverage to examine the implications of the FDI flows from different countries for environmental degradation in recipient countries. Secondly, a future study could cover recent years to investigate whether the impact of FDI flows from other countries had different effects in more recent periods. Thirdly, even though this paper examines the FDI flows from each OECD country to BRICS countries, the FDI flows data could further be disaggregated to examine the implications of the FDI flows to different sectors. For instance, Cansino et al. (2021) examined Spanish FDI flows to various industries and found that the pollution haven hypothesis is not confirmed when aggregate FDI flows from Spain are used, but PHH is confirmed in primary and manufacturing sectors. Therefore, a future study could examine whether the FDI flows to different sectors from developed to developing countries result in environmental degradation in the recipient country or not.

## Data Availability

The data for all the variables, except bilateral FDI flows, are obtained from the World Development Indicators (World Bank 2021). The bilateral FDI flows from eleven OECD to BRICS countries are obtained from OECD (2021). All the data used is publicly available from these two sources.

## Footnotes

1 To obtain a balanced data set, if FDI flows from OECD countries to BRICS countries were missing in a given period, average FDI flows between preceding and succeeding years are used to interpolate the missing data.
